# First trimester prediction of gestational diabetes mellitus by machine learning in twin pregnancies

**DOI:** 10.1007/s00404-025-08262-6

**Published:** 2026-01-20

**Authors:** Yoram Louzoun, Tamar Michelson, Mar Bennasar, Ran Svirsky, Elisa Bevilacqua, Nadav Kugler, Karl Kagan, Richard Nicholas Brown, Heidy Portillo Rodriguez, Anna Goncé, Antoni Borrell, Julia Ponce, Annegret Geipel, Adeline Walter, Corinna Simonini, Brigitte Strizek, Tanja Lennartz, Armin Bauer, Federica Meli, Eleonora Torcia, Adi Sharabi-Nov, Ron Maymon, Kypros H. Nicolaides, Hamutal Meiri

**Affiliations:** 1 Department of Mathematics, Bar Ilan University, 5290002 Ramat Gan, Israel; 2 Hospital Clinic de Barcelona, 08036 Barcelona, Spain; 3 Department of Obstetrics and Gynecology, Shamir (Assaf Harofeh) Medical Center, Zerifin, Israel; 4 Medical Genetic Unit, Department of Obstetrics and Gynecology, Samson Assuta Ashdod University Hospital, Ashdod, Israel; 5 Department of Women and Child Health, Women Health Area, Fondazione Policlinic Universitario Agostino Gemelli IRCCS, Rome, Italy; 6 Maternal Fetal Medicine Unit, Department of Obstetrics and Gynecology, University Hosp Tubingen, Tubingen, Germany; 7 Division of Maternal Fetal Medicine, Department of Obstetrics & Gynaecology, McGill University Health Centre, Montreal, Canada; 8 Department of Obstetrics and Prenatal Medicine, University Hospital Bonn, Bonn, Germany; 9 Department of Statistics, Tel Hai Academic College, Tel Hai, Qiryat Shemona, Israel; 10 Faculty of Medicine, Tel Aviv University, Tel Aviv, Israel; 11 The Fetal Medicine Research Center, King’s College Hospital, London, UK; 12 Faculty of Health Sciences and Medicine, Ben-Gurion University of the Negev, Be’er Sheva, Israel; 13 Department of Statistics, Ziv Medical Center, Safed, Israel

**Keywords:** Twin pregnancy, Machine learning, Prediction of GDM, Screening markers

## Abstract

**Introduction:**

We aimed to develop a machine learning model for first-trimester prediction of gestational diabetes mellitus (GDM) in twin pregnancies using a prospective international, multi-center cohort and identify useful predictive markers.

**Methods:**

Pregnant women with two live fetuses were enrolled at 11 + 0 to 13 + 6 weeks’ gestation and followed until delivery. GDM was diagnosed at 24–28 weeks’ gestation using the two-stage GCT and OGTT tests. Biochemical, biophysical, and blood assessments were conducted at three periods during pregnancy. Multiple machine learning models evaluated demographic, clinical, and laboratory parameters, including maternal factors (BMI, age, medical history), sonographic markers (crown rump length, estimated fetal weight, uterine artery pulsatility index), and blood and biochemical markers (placental growth factors, blood glucose, cell counts). LightGBM, XGBoost, and logistic regression models were compared using area under the curve (AUC) analysis.

**Results:**

Among 596 women, 99 (16.6%) developed GDM. LightGBM demonstrated superior performance (AUC = 0.72, 95% CI 0.69–0.75). First-trimester high BMI was the strongest predictor, followed by elevated white blood cell counts and platelet levels. Detection rates (DR) were 28% and 42% at 10% and 20% false positive rates (FPR), respectively. Previous GDM was associated with an increased risk for GDM.

**Discussion:**

GDM in twins is associated with certain characteristics of the first-trimester. Information from later trimesters has a limited impact. The GDM probability risk score increased with the severity of the treatment. An app to predict this score is available at: twin-pe.math.biu.ac.il.

**Supplementary Information:**

The online version contains supplementary material available at 10.1007/s00404-025-08262-6.

## What does this study add to the clinical work

**Table:** 

This study demonstrates that machine learning models can achieve good predictive performance (AUC = 0.72) for gestational diabetes mellitus (GDM) in twin pregnancies using readily available first-trimester clinical parameters, with BMI, white blood cell counts, and platelet levels emerging as key predictors. The approach enables early risk stratification in twin pregnancies, potentially allowing for earlier interventions and improved maternal-fetal outcomes in this high-risk population.	

## Introduction

Gestational diabetes mellitus (GDM) is glucose intolerance or hyperglycaemia that is first recognized or appears during pregnancy, and can result in short-and long-term adverse outcomes for women and their newborns, including diabetes and obesity [1–4]. With an increasing prevalence worldwide, there is a need to assess strategies, including nutritional intervention, therapeutic intervention, and additional lifestyle changes that might prevent the development of GDM [3, 4]. GDM is one of the main origins of life morbidity and mortality developed during the first nine months in-uterus. Growing within a mother with GDM shapes the offspring’s life response to glycemia and metabolic stress and is considered a major cause for adulthood obesity, diabetes, and cardiovascular diseases.

The main approach for GDM diagnosis is the 24–28 weeks’ gestation testing of an elevated glucose challenge test (GCT) and/or oral glucose tolerance test (OGTT) [1]. Positive participants are treated either by nutritional intervention or anti-diabetic medications (mainly insulin, glyburide, and metformin [3–6]. Regardless of the unequivocal results of the HAPO study, the two-step approach predating the HAPO study is still widely used for diagnosing GDM [7, 8].

Yet, several randomized studies and meta-analyses have shown that partial risk stratification in the first trimester enables the beginning of preventive measures that may improve both maternal and neonatal outcomes in singleton pregnancies with and without maternal obesity [9, 10]. Former studies revealed that features recorded at enrolment, such as obesity, South/East Asian ethnicity, GDM in previous pregnancies, and a family history of GDM, are useful features in early GDM prediction [11]. Other studies indicated that current first-trimester biophysical and biochemical markers used to predict the risk of developing preeclampsia can also be used in stratifying the risk of developing GDM [12]. These are further improved by measuring blood biochemical tests of glucose levels after overnight fasting [13]. Other identified soluble CD163, placental protein 13, and tumor-necrosis factor alpha as promising markers [13]. Increased nuchal translucency (NT) was also suggested as a useful biomarker [14].

GDM in twin pregnancies is underrepresented in the clinical literature, and the incidence rate has not been uniformly reported [15]. Some studies have found twin pregnancies to be at a higher risk for GDM, while others have found similar rates as in singleton pregnancies (20–22). Regardless of many differences in the features, the rate of GDM-associated complications is associated with certain different maternal factors, such as advanced maternal age in pregnancies, a higher incidence of conception by assisted reproduction technologies (ART), among others. The cutoffs for GCT and OGTT test results are similar in GDM diagnostics of singletons and twin pregnancies, the control of the hyperglycemic index is similarly managed by nutritional intervention, and the dose of insulin and metformin administered in twins is the same as in singletons when such treatment is selected [16, 17].

First-trimester prediction of GDM in singletons by machine learning was found to be useful [18]. Here, we applied machine learning for predicting GDM in twin pregnancies using longitudinal features, recorded at enrolment, first, second, and third trimester. These markers are already in use to predict preeclampsia and inflammation. The study is an exploratory evaluation of any marker available in our comprehensive database.

## Materials and methods

### Study design and participants

This analysis is a part of the Pre-Twin Screen study funded by EP PerMed (project # JTC2019-61) to develop a model of multi-markers, personalized, prenatal diagnostics to predict feto-maternal complications in twin pregnancies [19]. Enrolment started in December 2020 and ended in August 2023. Women with two live monochorionic diamniotic (MCDA) and dichorionic (DC) twins at 11 + 0–13 + 6 weeks’ gestation, calculated from the crown-rump length (CRL) of the larger fetus [20] were enrolled. The inclusion criteria were women delivering two live, non-malformed neonates > 24 weeks’ gestation. These criteria were fulfilled by 596 women: 75 from Rome, Italy**,** 75 from Montreal, Canada, 93 from Barcelona, Spain, 99 from Tubingen, and 141 from Bonn, Germany, and 113 from Zerifin, Israel (Table 1).

**Table 1:** Patient characteristics

Field	All	Unaffected	GDM	*p* values
	(*n* = 596)	(*n* = 497)	(*n* = 99)	
Maternal characteristics at enrolment				
GA, Me (IQR)	36.6 (34.9–37.3)	36.4 (34.9–37.3)	37 (3.55 -3.74)	0.080
MA, yr. Me (IQR)	34.3 (31.0–37.3)	34.2 (31.0–37.3)	35.0 (31.1–37.4)	0.403
BMI, Kg/h2, me (IQR)	24.2 (21.2–28.4)	23.9 (21.0–27.6)	27.0 (22.1–30.6)	**0.0001**
Nulliparous, *n* (%)	298 (50.00)	245 (49.30)	53 (53.54)	0.509
Ethnicity white, *n* (%)	523 (87.75)	441 (88.73)	82 (82.83)	0.129
Conception spontaneous, *n* (%)	350 (58.72)	297 (59.76)	53 (53.54)	0.265
Chronic medical disease, *n* (%)				
Diabetes meletus	9 (1.51)	6 (1.21)	3 (3.03)	0.176
Chronic hypertension	13 (2.18)	10 (2.01)	3 (3.03)	0.462
Hypothyroidism	63 (10.57)	52 (10.46)	11 (11.11)	0.858
Previous GDM	11 (1.85)	5 (1.01)	6 (6.06)	**0.005**
Chorionicity, *n* (%)				
DCDA	427 (71.64)	351 (70.62)	76 (76.77)	0.272
MCDA	169 (28.36)	146 (29.38)	23 (23.23)	0.272
Maternal characteristics at delivery				
GA, me (IQR)	36.6 (34.9–37.3)	36.4 (34.9–37.3)	37 (35.5–37.4)	0.080
GA < 37, *n* (%)	329 (55.20)	283 (56.94)	46 (46.46)	0.060
GA < 34, *n* (%)	87 (14.60)	76 (15.29)	11 (11.11)	0.350
Cesarean delivery, *n* (%)	402 (67.45)	337 (67.81)	65 (65.66)	0.725
Preeclampsia, *n* (%)	66 (11.07)	54 (10.87)	12 (12.12)	0.726
MAP mmHg, Me (IQR)	92 (85.6–99)	92 (85.6–99)	93.1 (84.7–99.8)	0.965
Treatment, *n* (%)				
Insulin	24	0	24	
Metformin	7	0	7	
Nutritional intervention	25	0	25	
Newborn characteristics				
Field	All (*n* = 1192)	Unaffected (*n* = 994)	GDM (*n* = 198)	*P*
Delivery—newborn g, Me (IQR)	1192 (100.00)	994 (100.00)	198 (100.00)	1.000
Birthweight A, g, Me (IQR)	2425 (2105–2700)	2420 (2077–2675)	2527.5 (2150.0–2770.0)	0.074
Birthweight B, g, Me (IQR)	2380 (2052–2670)	2380 (2040–2670)	2370 (2172–2691)	0.227
Weight < 2500 g, *n* (%)	423 (70.97)	355 (71.43)	68 (68.69)	0.628
Both twins	261 (43.79)	223 (44.87)	38 (38.38)	0.268
One twin	162 (27.18)	132 (26.56)	30 (30.30)	0.459
FGR (10-centile)	144 (12.08)	119 (11.97)	25 (12.6)	0.095
Female newborn, *n* (%)	595 (49.92)	492 (49.50)	103 (52.02)	0.534
APGAR 1 min. < 7, *n* (%)	220 (18.46)	191 (19.22)	29 (14.65)	0.160
APGAR 5 min. < 7, *n* (%)	122 (10.23)	110 (11.07)	12 (6.06)	0.039
NICU (days), Me (IQR)	12 (4–22.5)	12 (3.75–22.5)	13 (6.25–25)	0.915
NICU admission, *n* (%)	272 (22.82)	237 (23.84)	35 (17.68)	0.064
Stillbirth > 24 wks, *n* (%)	11 (0.92)	9 (0.90)	2 (1.00)	0.227

Significant differences are presented in bold letters

The master study ethical approval was obtained by the Shamir (Assaf Harofe) Medical Center (Trial # 0043-20-ASF) and the Israel Ministry of Health (# 202016632). It was subsequently endorsed in all other participating centres. All participants provided written informed consent. The protocol was registered in Clinicaltrials.gov with an ID #: NCT04595214.

### Investigations in the first trimester

At enrolment, we recorded maternal demographics, medical and pregnancy history, including maternal age, their BMI and ethnic origin, whether they had GDM in a previous pregnancy (for multipara participants), and their family history of GDM. We also entered features of the current pregnancy, including the mode of conception, chorionicity, among others [12, 14]. Blood cell counts, blood glucose levels after overnight fasting, and blood groups were determined from blood samples. Ultrasound was used to determine the NT width [19, 21]. Estimated fetal weight (EFW) was determined according to Hadlock et al. [22] using the four-parameter formula for measuring the biparietal diameter (BD), head (HC) and abdominal (AC) circumference, and femur length (FL) at any of 11–13, 20–22, 24–26, 28–30, 32–34 and 36–37 weeks’ gestation from each twin, unless the pregnancy had been delivered earlier. In MCDA twin pregnancies, additional ultrasound scans were carried out at 15–16 and 17–18 weeks’ gestation [23]. For this study, we used the values of 11–13, 20–22 and 32–33. The mean uterine arteries pulsatility index (UtA-PI) of the left and right uterine arteries was measured by transvaginal or transabdominal color Doppler ultrasound [24]. Mean arterial pressure (MAP) was evaluated by validated automated devices and a standardized protocol [25].

We measured the serum level of pregnancy-associated plasma protein A (PAPP-A), placental growth factor (PlGF), and soluble fms-like tyrosine kinase 1 (sFLT-1) by automated analyzers (Elecsys Analyzer, Roche Diagnostics International AG, Switzerland; Delfia Express, Revvity, Turku, Finland; or BRAHMS KRYPTOR compact PLUS, Thermo Fisher Scientific, Germany). Cell-free fetal DNA (cffDNA) fraction was determined as part of the examination of maternal blood by non-interventional prenatal testing (NIPT) to identify major trisomies [26].

### Investigations in the second and third trimesters

Except for CRL, NT, cffDNA fraction, and blood type, which were only determined in the first trimester, and GCT and OGTT, which are only measured once at gestational weeks 24–28, all values measured in the first trimester were also determined in the second and third trimesters. Ultrasound scans to identify malformations, blood cell counts, hemoglobin, blood biochemistry for glucose, iron, PlGF, sFLT-1, and PAPP-A were conducted in any of the 1st, 2nd, and 3rd trimesters as reported previously.

### Delivery data

Delivery data were extracted from the electronic medical records of participating hospitals, or by hospital discharge reports, and women’s phone interviews if delivery occurred outside the enrolling hospitals. The outcome measure was delivery with GDM. Preterm delivery (PTD) was defined as any delivery before 37 weeks’ gestation [27]. Values entered covers the entire process and mode of delivery, any test taken during the admission to delivery, newborn details, and NICU data, if required.

### The diagnosis of GDM

The diagnosis of GDM was conducted at 24–28 weeks of gestation according to the guidelines of the American College of Obstetrics and Gynecology [28], although with some slight local variations. First GCT (50 g) was conducted, and if above 200 mg/dL, results were considered positive. If values were > 140 but below 200 mg/dL, a secondary 100 g, OGTT was performed in the morning after overnight fasting. Women were considered positive if two out of four measurements were ≥ 95 (time zero), 180, 155, and 145 mg/dL, at the respective next 1, 2, or 3 h’s. In Barcelona, they followed the National Diabetes Data Group criteria [29] stipulate using fasting 105 mg/dL at time zero and 190 mg/dL, 165 mg/dL, and 145 mg/dL for 1,2, and 3 h, respectively. In patients where GCT or OGTT could not be accomplished, evaluation of blood glucose levels in the morning and 1 h after each meal was performed, and if values were pathological, a diagnosis of GDM was made. Women with GDM were treated with nutritional intervention, metformin, and insulin as necessary. Following the diagnosis of GDM, centers used nutritional intervention, insulin, metformin, or their combinations to improve outcomes after diagnosis, hoping to prevent GDM. Clinical management was according to the 24–28 testing of GCT and OGTT (excluding chronic diabetes).

### Machine learning and statistical methods

During the study, databases were shared with the data manager every month, and missing entries that were overlooked initially were subsequently completed from the source site records. As such, there were practically no missing data, and the few missing values were replaced by the median.

The data were converted into *Z*-scores, using the training set average and standard deviations. Categorical parameters were represented using one-hot encoding and were not normalized. For the prediction, we tested XGBoost [30], logistic regression, and Light Gradient Boosting Machine (Lgbm) [31]. For the logistic regression, a ridge regularization was used with a coefficient of 1.0. For the XGBoost, 50 trees were used, with a max depth of 4, gamma = 8, and eta = 1/3. For the Lgbm, 50 trees were used, with a learning rate of 0.1, a bagging fraction of 0.7 for both samples and features, and a limitation of at least 20 samples per leaf. Given the limited size of the sample, no hyperparameter tuning was performed in the main text. A similar analysis with hyperparameter optimization was performed and described in the supplementary material.

We evaluated for each woman four groups of variables: (1) demographics, and medical and obstetric history collected at the time of enrolment, (2–4) marker values measured at each of the three pregnancy trimesters. The association between the different features in unaffected participants compared to GDM patients was performed using the *p*-value of the Point-Biserial Correlation Coefficient, and the correlation coefficient among the different features [32].

In each trimester, and for each model, we used the cumulative information until this trimester. We divided the data 10 times randomly into 80% training and 20% test. We computed the area under the curve (AUC) of the receiver operating characteristic (ROC) curve for each split. In parallel, the predictions for all the tests were combined to produce a single ROC for all continuous variables.

Continuous patients’ characteristics are presented as medians with interquartile range (IQR), and compared by the Mann–Whitney *U*-test or Kruskal–Wallis non-parametric test. Categorical values are presented as *n* (%) and were compared using the Chi-square test or Fisher’s exact *U* test.

All estimates and statistical tests were performed using MATLAB version 2024a (MathWorks Inc., Natick, MA, USA). Power analysis was calculated with WinPepi software Ver. 11.65 (http://www.brixtonhealth.com/pepi4windows.html). Note that the data needed for the prediction is only available upon request and following the appropriate ethics approvals.

The machine learning prediction accuracy was measured either through the aggregation of all folds and the computation of a ROC curve, and the resulting AUC on the combined data. We also computed the average AUC on all folds. The same was done for estimating marker efficacy. The results in the main text are without class stratification and without hyperparameter optimization. We have also tested the same model with class stratification and hyper-parameter optimization using Optuna.

## Results

### Sample and patients’ characteristics

The analysis included 596 women of which 99 (16.6%) participants developed GDM, and half of the GDM participants (48 patients, 8.05%) delivered preterm (< 37 weeks’ gestation). The characteristics of the study population are summarized in Table 1. At enrolment, the women in the GDM group, compared with the unaffected pregnancies, had higher BMI, a greater proportion of women were nulliparous, and the median of MAP was higher. Note that the chronic diabetic cases were listed in Table 1 for cohort characterization but not included in in the machine learning analysis in Table 2.

**Table 2:** Markers values across trimesters (T)

Field	All	Unaffected	GDM	*p* values
CRL twin A mm, Me (IQR)	64.5 (57.8–71.0)	64.1 (57.6–70.7)	64.8 (58.1–72/0)	0.473
CRL twin B mm (IQR)	64.8 (57.4–70.9)	64.6 (57.5–70.5)	65.0 (57.2–72.0)	0.452
NT twin A, mm, Me (IQR)	1.6 (1.3–1.8)	1.6 (1.3–1.8)	1.5 (1.2–1.8)	0.761
NT twin B, mm, Me (IQR)	1.6 (1.3–1.9)	1.6 (1.3–1.9)	1.6 (1.4–2.0)	0.164
WBC (Cells × 1000/mL), Me (IQR)				
T1	9 (7.5–10.5)	8.9 (7.4–10.4)	9.8 (8.3–11.3)	**0.0006**
T2	9.7 (8.1–11.2)	9.6 (8.0–11.1)	10.1 (9.3–11.9)	**0.0005**
T3	9.5 (8.0–11.1)	9.5 (7.9–11.0)	10.1 (8.2–11.6)	0.209
Platelets (# × 1000//mL), Me (IQR)				
T1	247.0 (212.0–288.0)	243.0 (211.5–285.0)	268.5 (218.5–298.5)	**0.0169**
T2	242.0 (208.0–282.0)	241 (208–281)	251.0 (219.0–296.0)	0.274
T3	214.0 (180.0–258.0)	214 (180.2–225.9)	212.5 (179.5–248.0)	0.634
Glucose (mg/dL), Me (IQR)				
T1	79 (73–84.7)	78 (73–84.7)	80 (75.7–85)	**0.027**
T2	79 (72–87)	79 (72–86.1)	82 (75.5–90.1)	**0.008**
T3	77 (68–87.2)	76 (68–86)	80 (68.7–91.9)	0.140
EFW (grams),				
T1 twin A	63.8 (58.0–72.1)	63.9 (58.3–71.9)	63.4 (56.6–72.9)	0.483
T1 twin B	62.7 (57.0–69.5)	662.8 (57.4–69.5)	61.2 (55.3–69.6)	0.123
T2 twin A	406.0 (361.0–474.0)	402.0 (358.0–470.0)	424.5 (377.0–504.0)	**0.020**
T2 twin B	402.5 (355.0–461.0)	400.5 (355.0–457.0)	409.0 (361.0–477.0)	0.453
T3 twin A	1397.5 (1200.0–1888.0)	1399.5 (1203.0–1887.5)	1391.5 (1181.0–1888.0)	0.417
T3 twin B	1390.0 (1202.0–1893.5	1414.0 (1213.5–1905.0)	1323.0 (1141.0–1861.5)	**0.050**
UtA-PI. Me (IQR)				
T1	1.4 (1.1–1.7)	1.4 (1.1–1.7)	1.4 (1.2–1.7)	0.410
T2	0.8 (0.7–0.9)	0.8 (0.7–0.9)	0.8 (0.7–1.0)	0.222
T3	0.7 (0.6–0.8)	0.7 (0.6–0.8)	0.7 (0.6–0.8)	0.978

Significant differences are presented in bold letters

At delivery, MAP, urine protein, and liver enzymes were higher in the GDM group. Newborn birthweight was lower in the GDM group, and the group also had a higher incidence of NICU admission.

### GDM markers

**Fig. 1:**
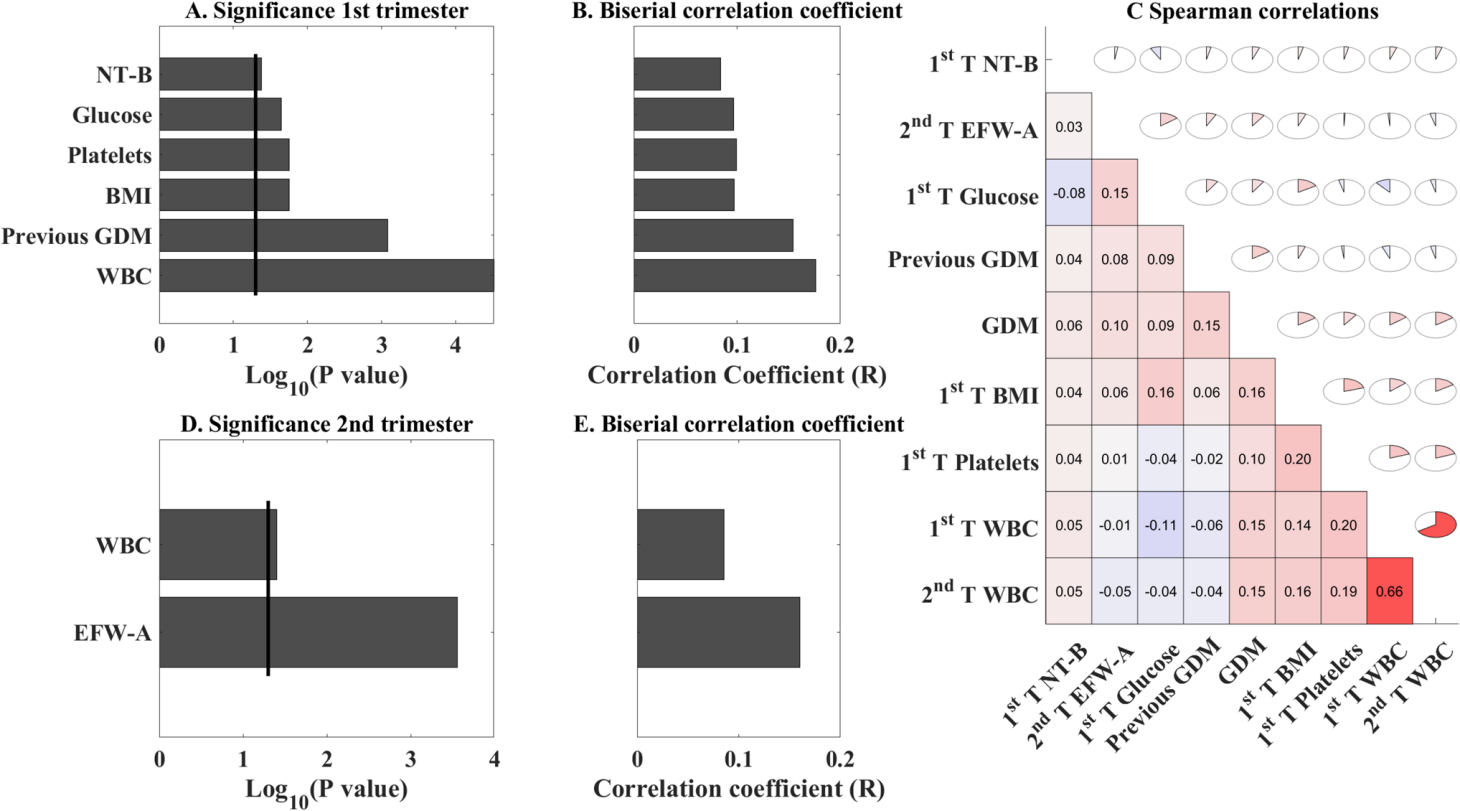
Significant markers and correlations. **A** Log p-value of two-point bi-serial correlation between features available at the first trimester (T1) according to whether they had a history of previous GDM. **B** Correlation coefficient R for the same test. **C** Spearman correlation coefficient between each pair of variables found to be significant in the Log p-value plot presented in A, marked by color and full fraction of the circle. **D**–**E** log p-value and the coefficient of the correlation of significant features from the second and third trimesters (T2, T3), indicating that the most effective features are available already in the first trimester.*NT* nucal translucency, *BMI* body mass index, *WBC* white blood cells. Only the values that were found to be different between GDM and unaffected participants are listed

The most effective first-trimester predictors of GDM development (Fig. 1A, B) were higher counts of white blood cells (WBC) and platelet levels, followed by higher BMI. A history of GDM in previous pregnancies also emerged as a significant risk factor for multiparous women. Notably, the blood glucose level of 100 g/dL after overnight fasting (standard normal level) was found to be a less effective marker compared to the above parameters.

We further tested the correlations between the different markers associated with GDM (Fig. 1C). The correlation analysis revealed positive associations between the most significant predicting markers and the development of GDM. Accordingly, the correlation matrix illustrates modest interconnections between predictive variables. The strongest correlations were observed between serial measurements of the same parameters across trimesters, particularly for WBC counts. Repeated biochemical testing of blood glucose levels after overnight fasting also had high correlations across trimesters. Second-trimester EFW and MAP were also demonstrated to be correlated with GDM diagnosis. Third-trimester characteristics were not useful.

### GDM prediction

Given these associations, we tested GDM prediction by first-trimester data. Of the three machine learning models, the LGBM consistently provided the highest AUC (0.72 (95% CI 0.69–0.75) vs. 0.66 (95% CI 0.63–0.69) in XGBoost and 0.59 (95% CI 0.54–0.62) in logistic regression, *p* < 0.05 for LGBM vs logistic regression using 100 training/test permutations.

Models using only ethnicity data demonstrated poor discriminative ability (AUC = 0.53, which is not significantly different than random).

**Fig. 2:**
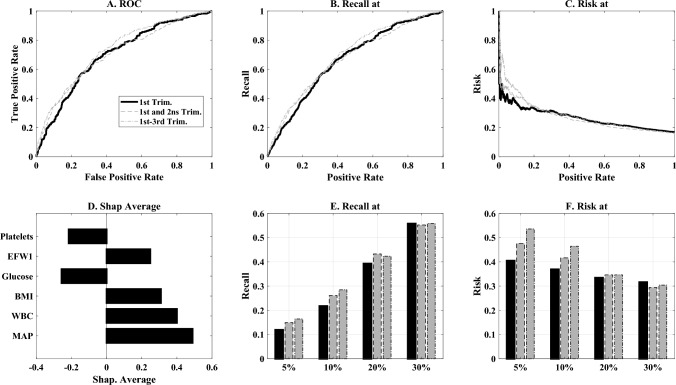
GDM prediction efficacy and risk stratification. **A** Receiver operation characteristic (ROC) curves for GDM prediction using information available at the first trimester (full black line), or in the first, second or third trimester (gray lines). **B** Recall as a function of a fraction of the population, defined to be positive for the same analysis. **C** Risk of developing GDM (fraction of women that have GDM, as a function of positive rate. **E**, **F** Recall and risk at 5,10,20 and 30% false positive rates (FPR). The black bars are the first trimester model, and the gray bars are the second and third trimesters models. **D** Average of Shapley factors for the first-trimester’s most effective markers

Incorporating markers measured in the first-trimester markedly improved the test set AUC (Fig. 2A), leading to an AUC of 0.68 (95% CI 0.64–0.71) by the LGBM model.

We tested whether information collected in the second and third trimesters improved the AUC, and found that it increased slightly to 0.**72** (95% CI 0.69–0.75) when second-trimester values were added. The addition of third-trimester data provided no further improvement in predictive accuracy (Fig. 2A).

Using the maternal features, first and second trimester model, at a false positive rate (FPR) of 10%, the detection rate was 28%, which increased to 42% when the FPR was set at 20% FPR (Fig. 2 E, F).

Women scoring in the top 10% of our risk prediction algorithm demonstrated greater than 40% probability of developing GDM (Fig. 2B and calibration plot in Supplementary Fig. 1). Conversely, those in the bottom 40% of the risk distribution had less than 5% GDM risk (Fig. 2C and Supplementary Fig. 1).

A Shapley analysis (Fig. 2D) highlights that the most effective markers are the first-trimester platelets, followed by BMI, MAP, EFW, and WBC. Ethnicity and first-trimester blood glucose of 100 mg/dL had a limited efficacy.

To ensure the stability of our results, we repeated the analysis with a stratified training/test division (Supplementary Fig. 2). Optimization on the hyperparameters (Supplementary Fig. 3), and analysis of each fold by itself (Supplementary Fig. 4). All these supplementary test methods have generated similar results to the ones described in the main text here.

**Fig. 3:**
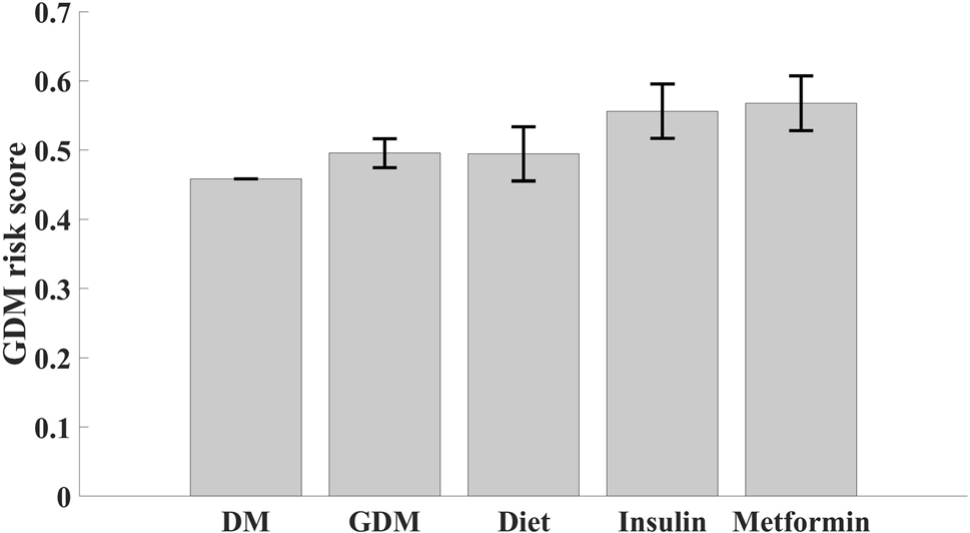
Prediction score and treatment. Mean (± standard deviations) of GDM prediction score for different groups on the test set. The score was computed using a model trained on the test set and then applied to each group in the test set. Chronic DM patients listed in Table 1 were not included in the machine learning analysis

Analysis of risk scores across treatment categories (Fig. 3) revealed a stepwise progression of predicted risk. Accordingly, untreated women exhibited the lowest average risk scores, followed by women managed only by nutritional intervention. Those requiring pharmacological intervention demonstrated progressively higher risk scores, with insulin-treated patients showing higher scores than those managed with metformin. This pattern suggests that our prediction algorithm not only identifies GDM risk but may also provide insight for adjusting the intervention for reaching improved outcomes after GDM diagnosis, hoping to prevent GDM according to risk severity.

## Discussion

This international, multi-center, and prospective study of 596 mothers with twin pregnancies represents the first comprehensive machine learning approach for longitudinal prediction of the risk of developing GDM, with a major “weight” offered by first-trimester parameters. The model demonstrated robust predictive capability for combined features recorded at enrolment and first-trimester variables. Medium prediction efficacy with AUC = 0.72 was achieved when novel predictive markers, mainly very simple test performed almost everywhere, were included beyond the traditional screening parameters. Second-trimester markers added slightly to the prediction efficacy. Third-trimester markers were not useful.

Our finding that first-trimester BMI constitutes a strong predictor of GDM in twin pregnancies aligns with findings from singleton pregnancies [33]. However, the prominence of hematological parameters—specifically WBC counts and platelet levels from both first and second trimesters—represents a novel observation in twin gestations, not previously reported. This association may reflect underlying inflammatory processes that precede clinical GDM manifestation. Emerging evidence suggests that low-grade inflammation contributes to the development of insulin resistance [34]. Our findings imply that these inflammatory markers may have particular relevance in the physiologically demanding context of twin pregnancies. This is interesting since a former study of Syngelaki et al [35] found that a major inflammatory marker—C Reactive protein (CRP)—is not an effective marker in singleton pregnancies, indicating that high levels of WBC may be a specific marker in twin pregnancies. It elevated the possibility of a separate bone marrow process underlying the increase in WBC is coccuring in twin compared to singleton pregnancies. It is worth noting that blood counts, platelet levels, and BMI are universally used in the majority of clinics, making our findings easier for implementation in predicting GDM in twins.

Periodic measurements of blood glucose level are used in the standard blood biochemical testing of pregnant women, and values > 100 mg/dL are considered for suspected development of GDM, although diagnosis is made according to the 24–28 tests of GCT and OGTT. Our analysis demonstrated that such blood glucose tests in the first and second trimesters in twin pregnancies have lower efficacy compared to elevated BMI, WBC, and platelet levels when used in our GDM prediction model. It suggests that the periodic blood sugar level testing may miss the capture of the risk of developing GDM in twin pregnancies. Interestingly, Hiersch et al [36] previously noted altered glucose metabolism patterns in twin versus singleton pregnancies, but did not propose a model to translate their observations into a clinical application.

The association between EFW and GDM development in the second trimester suggests that subtle alterations in fetal growth trajectories may precede formal GDM diagnosis and are consistent with an early onset of GDM and GDM Diagnosis [37]. In fact, Sovio et al [38] already showed that in singleton pregnancies, increased fetal growth velocity preceded GDM diagnosis. Our study extends this concept to twin gestations, highlighting potential opportunities for earlier intervention.

Current GDM screening in twin pregnancies follows protocols developed for singleton pregnancies after the HAPO study, typically employing either the one-step (75 g OGTT) or two-step approach (50 g GCT followed by 100 g OGTT) between 24–28 weeks of gestation [38]. However, this timeline may be sub-optimal for twin pregnancies, given that almost 50% of twin pregnancies are delivered preterm, which translates into a very short period (maximum 10 but most frequently only 5–6 weeks) that is left for the preventive effect. Our model achieved an AUC of 0.67 using only first-trimester data, suggesting that some risk stratification could be implemented much earlier in pregnancy, not necessarily by any of these tests.

The negligible improvement in predictive accuracy when adding third-trimester data emphasizes that GDM pathophysiology in twin pregnancies is largely established by mid-pregnancy, or alternatively, that it is effectively regulated by nutritional intervention and medications. This challenges the conventional screening timeline and supports Cooray et al. [39] call for earlier assessment of the risk of developing GDM in high-risk pregnancies. While there is a debate whether twin pregnancies have a higher incidence of GDM versus singletons, the ability to identify 60% of GDM participants with virtually no false positives using first and second-trimester data, as we found out here, provides compelling evidence for implementing our risk-stratified screening approaches in the clinical management of twin pregnancies.

Furthermore, our model’s capacity to identify women in the lowest 40% of risk scores who have low GDM risk could substantially reduce unnecessary testing. Conversely, identifying women in the highest 10% of risk scores with > 40% probability of developing GDM enables targeted prophylactic interventions. Similar risk-stratification approaches may be achieved in singleton pregnancies [40]. Our study provides the first evidence supporting the necessity to apply the approach in twin gestations.

The step-wise progression of predicted risk scores across treatment categories (untreated → nutritional intervention → insulin → metformin) suggests that our model captures not only GDM occurrence but also risk severity, and it allows for the adjustment of the suitable intervention with nutrition intervention, insulin, metformin, or their combinations to obtain the best prevention, as was already proposed by Benhalima et al. [41] in singletons.

## Limitations

There are minor diversions in the GDM definition among participating centers derived to country guidelines. The ethics committee did not approve deviations from these local guidelines. Since multi-national studies are favorable for global implications, such minor differences have to be taken into consideration. However, it appears that these minor modifications add to the robustness of the model, showing that the results are not sensitive to the relatively minor differences in the precise definition.

Our study had 99 GDM cases and 497 unaffected cases. It is smaller than singleton cohorts, with statistical power to detect a difference from the null hypothesis (AUC = 0.5) above 99% at α = 0.01.

The modest contribution of ethnicity data to the predictive accuracy (AUC = 0.53) is in contrast with some singleton pregnancy studies [42, 43]. This may reflect the low fraction of non-whites in our cohort. Larger studies with more diverse populations are warranted to clarify these findings.

## Conclusions

This first longitudinal prospective multi-center study of GDM prediction in twins by machine learning provides a fair prediction of this major obstetric complication. A special role for inflammatory markers was discovered, previously not reported in singletons. Additional markers may add to the prediction efficacy. In addition, a correlation between the level of risk and the desired intervention was established.

## Supplementary Information

Below is the link to the electronic supplementary material.

Supplementary file1 (TIFF 439 KB)

Supplementary file2 (TIFF 430 KB)

Supplementary file3 (TIFF 294 KB)

Supplementary file4 (TIFF 272 KB)

## Data Availability

The data that support the findings of this study are not publicly available due to frequent cyber-attacks on hospitals and universities in Israel, but are available by communication with Prof. Yoram Louzoun at [louzo@math,BIU.AC](mailto:louzo@math,BIU.AC) for the dataset used for this analysis. The full study database is held by Dr. Nadav Kugler at [nadavkuglet@gmail.com](mailto:nadavkuglet@gmail.com), but is restricted by the ethics committee to use by applicants who can prove sufficient firewall protection of their computers. The use of the model is free to anyone by connecting to [twin-pe.math.biu.ac.il](http:/twin-pe.math.biu.ac.il).

## References

[1] Hod M, Kapur A, Sacks DA, Hadar E, Agarwal M, Di Renzo GC, Roura LC, McIntyre HD, Morris JL, Divakar H (2015) The international federation of gynecology and obstetrics (FIGO) initiative on gestational diabetes mellitus: a pragmatic guide for diagnosis, management, and care. Int J Gynaecol Obstet 131:S173–S211 29644654 10.1016/S0020-7292(15)30033-3

[2] Group GD (2008) Management of diabetes from preconception to the postnatal period: summary of NICE guidance. BMJ 336(7646):714–717 18369227 10.1136/bmj.39505.641273.AD PMC2276266

[3] Koning SH, Hoogenberg K, Scheuneman KA, Baas MG, Korteweg FJ, Sollie KM, Schering BJ, van Loon AJ, Wolffenbuttel BHR, van den Berg PP, Lutgers HL (2016) Neonatal and obstetric outcomes in diet- and insulin-treated women with gestational diabetes mellitus: a retrospective study. BMC Endocr Disord 16:52–62. 10.1186/s12902-016-0136-4 27680327 10.1186/s12902-016-0136-4 PMC5041294

[4] Brown J, Grzeskowiak L, Williamson K, Downie MR, Crowther CA (2017) Insulin for the treatment of women with gestational diabetes. Cochrane Database Syst Rev. 10.1002/14651858.CD012037.pub2 29103210 10.1002/14651858.CD012037.pub2 PMC6486160

[5] World Health Organization (2024) Diagnostic criteria and classification of hyperglycaemia first detected in pregnancy. https://apps.who.int/iris/handle/10665/85975 24199271

[6] Tieu J, Shepherd E, Middleton P, Crowther CA (2017) Dietary advice interventions in pregnancy for preventing gestational diabetes mellitus. Cochrane Database Syst Rev 3(1):CD006674. 10.1002/14651858.CD006674.pub3 10.1002/14651858.CD006674.pub3 PMC6464792 28046205

[7] Coustan DR, Lowe LP, Metzger BE, Dyer AR (2010) The hyperglycemia and adverse pregnancy outcome (HAPO) study: paving the way for new diagnostic criteria for gestational diabetes mellitus. Am J Obstet Gynecol 202(6):654-e1 10.1016/j.ajog.2010.04.006 PMC2897007 20510967

[8] Sacks DA, Hadden DR, Maresh M, Deerochanawong C, Dyer AR, Metzger BE, Lowe LP, Coustan DR, Hod M, Oats JJN (2012) Frequency of gestational diabetes mellitus at collaborating centers based on IADPSG consensus panel–recommended criteria: the hyperglycemia and adverse pregnancy outcome (HAPO) study. Diabetes Care 35(3):526–528 22355019 10.2337/dc11-1641 PMC3322716

[9] Calancie L, Brown MO, Choi WA, Caouette JL, McCann J, Nam EY, Werner EF (2025) Systematic review of interventions in early pregnancy among pregnant individuals at risk for hyperglycemia. Am J Obstet Gynecol MFM 7(3):101606. 10.1016/j.ajogmf.2025.101606 39788428 10.1016/j.ajogmf.2025.101606 PMC11885049

[10] Buelo AK, Kirk A, Lindsay RS, Jepson RJ (2019) Exploring the effectiveness of physical activity interventions in women with previous gestational diabetes: a systematic review of quantitative and qualitative studies. Prev Med Rep 14:100877. 10.1016/j.pmedr.2019.100877 31110933 10.1016/j.pmedr.2019.100877 PMC6510702

[11] Artzi NS, Shilo S, Hadar E, Rossman H, Barbash-Hazan S, Ben-Haroush A, Balicer RD, Feldman B, Wiznitzer A, Segal E (2020) Prediction of gestational diabetes based on nationwide electronic health records. Nat Med 26(1):71–76. 10.1038/s41591-019-0724-8 31932807 10.1038/s41591-019-0724-8

[12] Syngelaki A, Wright A, Gomez Fernandez C, Mitsigiorgi R, Nicolaides KH (2025) First-trimester prediction of gestational diabetes mellitus based on maternal risk factors. BJOG. 10.1111/1471-0528.18110 40000426 10.1111/1471-0528.18110 PMC12051238

[13] Gabbay-Benziv R, Doyle LE, Blitzer M, Baschat AA (2015) First trimester prediction of maternal glycemic status. J Perinat Med 43:283–289 25153547 10.1515/jpm-2014-0149

[14] Savvidou MD, Syngelaki A, Muhaisen M, Emelyanenko E, Nicolaides KH (2012) First trimester maternal serum free β-human chorionic gonadotropin and pregnancy-associated plasma protein A in pregnancies complicated by diabetes mellitus. BJOG 119(4):410–416 22324916 10.1111/j.1471-0528.2011.03253.x

[15] Rauh-Hain JA, Rana S, Tamez H, Wang A, Cohen B, Cohen A, Brown F, Ecker JL, Karumanchi SA, Thadhani R (2009) Risk for developing gestational diabetes in women with twin pregnancies. J Matern Fetal Neonatal Med 22(4):293–299 19340713 10.1080/14767050802663194

[16] Buerger O, Elger T, Varthaliti A, Syngelaki A, Wright A, Nicolaides KH (2021) First-trimester screening for gestational diabetes mellitus in twin pregnancies. J Clin Med 10(17):3814 34501262 10.3390/jcm10173814 PMC8432220

[17] Hiersch L, Berger H, Okby R, Ray JG, Geary M, Mcdonald SD, Murry-Davis B, Riddell C, Halperin I, Hasan H (2018) Incidence and risk factors for gestational diabetes mellitus in twin versus singleton pregnancies. Arch Gynecol Obstet 298:579–587 29971559 10.1007/s00404-018-4847-9

[18] Zaky H, Fthenou E, Srour L, Farrell T, Bashir M, El Hajj N, Alam T (2025) Machine learning based model for the early detection of gestational diabetes mellitus. BMC Med Inform Decis Mak 25(1):130 40082942 10.1186/s12911-025-02947-3 PMC11905636

[19] Meiri H, Kugler N, Svirsky R, Kagan O, Brown RN (2020) Pre-twin screen-a multi-disciplinary approach for a personalized prenatal diagnostics and care for twin pregnancies. Int J Womens Health Wellness 6(110):1353–2474

[20] Chaudhuri K, Su LL, Wong PC, Chan YH, Choolani MA, Chia D, Biswas A (2013) Determination of gestational age in twin pregnancy: which fetal crown–rump length should be used? J Obstet Gynaecol Res 39(4):761–765 23279688 10.1111/j.1447-0756.2012.02054.x

[21] Sweeting AN, Wong J, Appelblom H, Ross GP, Kouru H, Williams PF, Sairanen M, Hyett JA (2018) A first trimester prediction model for gestational diabetes utilizing aneuploidy and pre-eclampsia screening markers. J Matern Fetal Neonatal Med 31(16):2122–2130 28562122 10.1080/14767058.2017.1336759

[22] Hadlock FP (1990) Sonographic estimation of fetal age and weight. Radiol Clin North Am 28(1):39–50 2404304

[23] Meiri H, Kugler N, Sharon N, Svirsky R, Brown R, Portillo Rodriguez H, Goncé A, Bennasar M, Borrell A, Ponce J, Geipel A, Walter A, Simonini C, Bevilacqua E, Romanzi F, Kagan KO, Lennartz T, Bauer A, Maymon R, Nicolaides KH, Louzon Y, the Pre-Twin Screen Consortium (2025) Multi-center prospective study of fetal growth in twins consistent with the FMF growth curve. Obstet Gynecol Res 8(2):79–90. 10.26502/ogr0182

[24] Ridding G, Hyett JA, Sahota D, McLennan AC (2015) Assessing quality standards in measurement of uterine artery pulsatility index at 11 to 13+ 6 weeks’ gestation. Ultrasound Obstet Gynecol 46(3):299–305 25412757 10.1002/uog.14732

[25] Poon LCY, Kametas NA, Valencia C, Chelemen T, Nicolaides KH (2011) Hypertensive disorders in pregnancy: screening by systolic diastolic and mean arterial pressure at 11–13 weeks. Hypertens Pregnancy 30(1):93–107 20818956 10.3109/10641955.2010.484086

[26] Gil MM, Galeva S, Jani J, Konstantinidou L, Akolekar R, Plana MN, Nicolaides KH (2019) Screening for trisomies by cfDNA testing of maternal blood in twin pregnancy: update of the fetal medicine foundation results and meta-analysis. Ultrasound Obstet Gynecol 53(6):734–742 31165549 10.1002/uog.20284

[27] Goldenberg RL, Culhane JF, Iams JD, Romero R (2008) Epidemiology and causes of preterm birth. Lancet 371(9606):75–84. 10.1016/S0140-6736(08)60074-4 18177778 10.1016/S0140-6736(08)60074-4 PMC7134569

[28] Bulletins-Obstetrics C (2018) ACOG practice bulletin no. 190: gestational diabetes mellitus. Obstet Gynecol 131(2):e49–e64 29370047 10.1097/AOG.0000000000002501

[29] Ong KL, Stafford LK, McLaughlin SA, Boyko EJ, Vollset SE, Smith AE, Dalton BE, Duprey J, Cruz JA, Hagins H (2023) Global, regional, and national burden of diabetes from 1990 to 2021, with projections of prevalence to 2050: a systematic analysis for the global burden of disease study 2021. Lancet 10.1016/S0140-6736(23)01301-6 PMC10364581 37356446

[30] Chen T, Guestrin C (2016) Xgboost: A scalable tree boosting–system. In: Proceedings of the 22nd Acm sigkdd international conference on knowledge discovery and data mining, pp 785–794

[31] Ke G, Meng Q, Finley T, Wang T, Chen W, Ma W, Ye Q, Liu TY (2017) Lightgbm: a highly efficient gradient boosting decision tree. Adv Neural Inf Process Syst 30

[32] Tate RF (1954) Correlation between a discrete and a continuous variable. point-biserial correlation. Ann math stat 25(3):603–607

[33] Zhang C, Rawal S, Chong YS (2016) Risk factors for gestational diabetes: is prevention possible? Diabetologia 59(7):1385–1390 27165093 10.1007/s00125-016-3979-3 PMC6364673

[34] Pantham P, Aye ILMH, Powell TL (2015) Inflammation in maternal obesity and gestational diabetes mellitus. Placenta 36(7):709–715 25972077 10.1016/j.placenta.2015.04.006 PMC4466145

[35] Syngelaki A, Visser GHA, Krithinakis K, Wright A, Nicolaides KH (2016) First trimester screening for gestational diabetes mellitus by maternal factors and markers of inflammation. Metabolism 65(3):131–137 26892524 10.1016/j.metabol.2015.10.029

[36] Hiersch L, Berger H, Okby R, Ray JG, Geary M, McDonald SD, Murray-Davis B, Riddell C, Halperin I, Hasan H (2019) Gestational diabetes mellitus is associated with adverse outcomes in twin pregnancies. Am J Obstet Gynecol 220(1):102-e1 10.1016/j.ajog.2018.10.027 30595142

[37] Jin D, Rich-Edwards JW, Chen C, Huang Y, Wang Y, Xu X, Liu J, Liu Z, Gao Y, Zou S (2020) Gestational diabetes mellitus: predictive value of fetal growth measurements by ultrasonography at 22–24 weeks: a retrospective cohort study of medical records. Nutrients 12(12):3645 33260833 10.3390/nu12123645 PMC7760346

[38] Sovio U, Murphy HR, Smith GCS (2016) Accelerated fetal growth prior to diagnosis of gestational diabetes mellitus: a prospective cohort study of nulliparous women. Diabetes Care 39(6):982–987 27208333 10.2337/dc16-0160

[39] Cooray SD, Boyle JA, Soldatos G, Wijeyaratne LA, Teede HJ (2019) Prognostic prediction models for pregnancy complications in women with gestational diabetes: a protocol for systematic review, critical appraisal and meta-analysis. Syst Rev 8:1–10 31711547 10.1186/s13643-019-1151-0 PMC6844063

[40] Yamamoto JM, Kellett JE, Balsells M, Garcia-Patterson A, Hadar E, Sola I, Gich I, van der Beek EM, Castaneda-Gutierrez E, Heinonen S (2018) Gestational diabetes mellitus and diet: a systematic review and meta-analysis of randomized controlled trials examining the impact of modified dietary interventions on maternal glucose control and neonatal birth weight. Diabetes Care 41(7):1346–1361 29934478 10.2337/dc18-0102

[41] Benhalima K, Van Crombrugge P, Moyson C, Verhaeghe J, Vandeginste S, Verlaenen H, Vercammen C, Maes T, Dufraimont E, De Block C, Jacquemyn Y, Mekahli F, De Clippel K, Van Den Bruel A, Loccufier A, Laenen A, Minschart C, Devlieger R, Mathieu C (2020) Estimating the risk of gestational diabetes mellitus based on the 2013 WHO criteria: a prediction model based on clinical and biochemical variables in early pregnancy. Acta Diabetol 57:661–671 31915927 10.1007/s00592-019-01469-5

[42] Kawai VK, Levinson RT, Adefurin A, Kurnik D, Collier SP, Conway D, Stein CM (2017) A genetic risk score that includes common type 2 diabetes risk variants is associated with gestational diabetes. Clin Endocrinol (Oxf) 87(2):149–155 28429832 10.1111/cen.13356 PMC5533106

[43] Pinto Y, Frishman S, Turjeman S, Eshel A, Nuriel-Ohayon M, Shrossel O, Ziv O, Walters W, Parsonnet J, Ley C (2023) Gestational diabetes is driven by microbiota-induced inflammation months before diagnosis. Gut 72(5):918–928 36627187 10.1136/gutjnl-2022-328406 PMC10086485

