# Supplementary figures and images for: First trimester prediction of gestational diabetes mellitus by machine learning in twin pregnancies
Source: Arch Gynecol Obstet. 2026 Jan 20;313(1):52. doi: 10.1007/s00404-025-08262-6 (PMC12819435; doi:10.1007/s00404-025-08262-6)

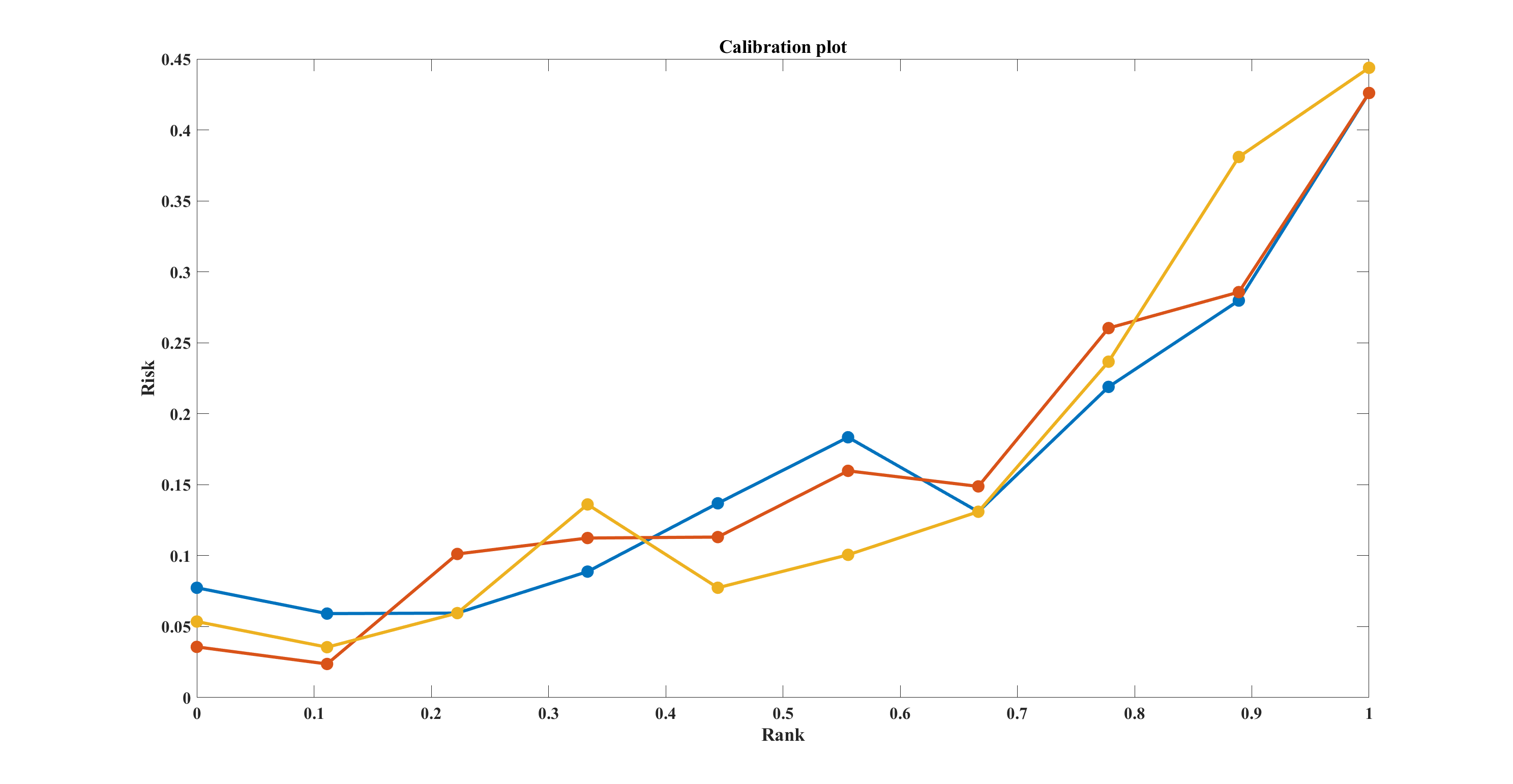

Supplement: Supplementary file 1 — Supplementary file1 (TIFF 439 KB) [file 404_2025_8262_MOESM1_ESM.tiff]

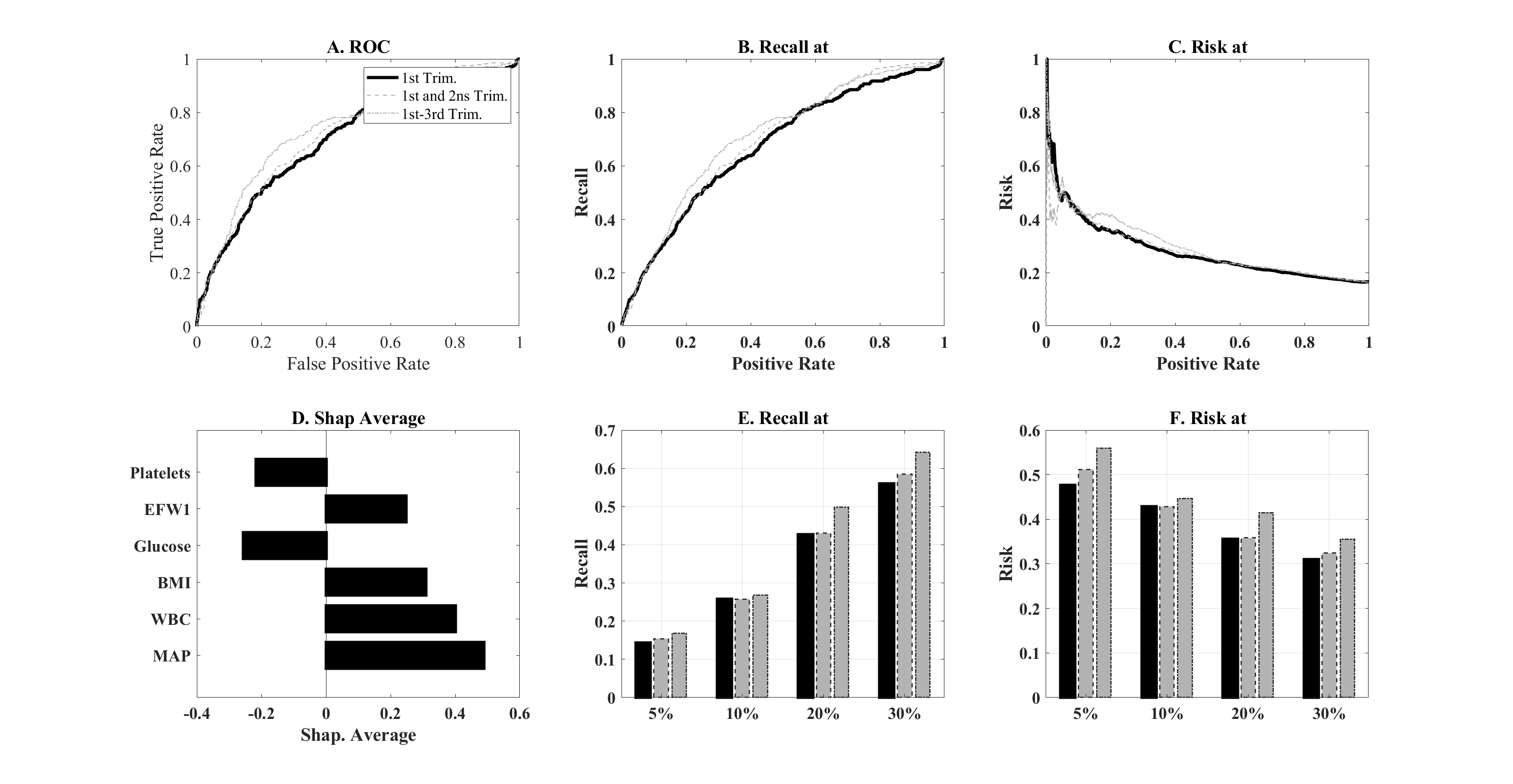

Supplement: Supplementary file 2 — Supplementary file2 (TIFF 430 KB) [file 404_2025_8262_MOESM2_ESM.tiff]

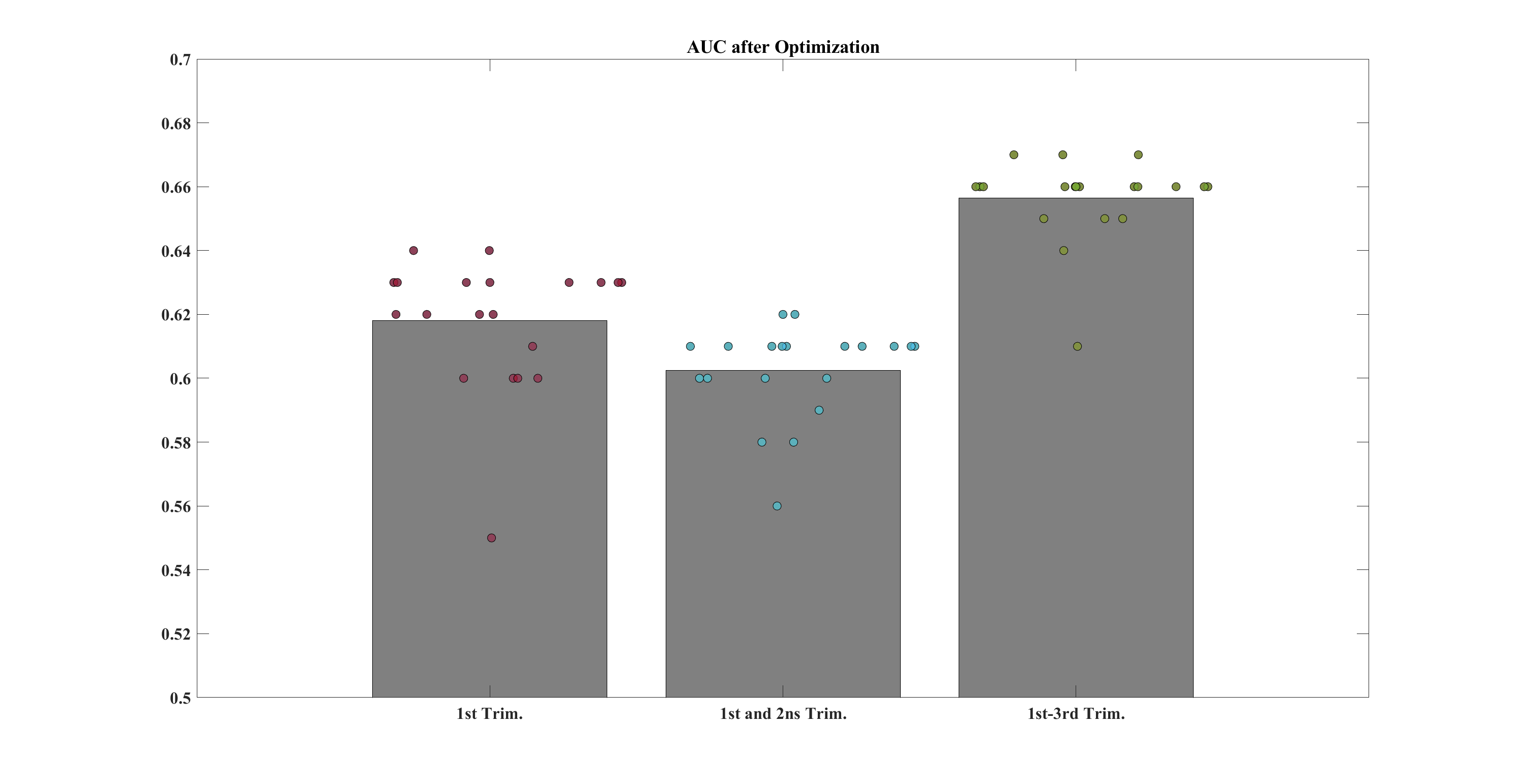

Supplement: Supplementary file 3 — Supplementary file3 (TIFF 294 KB) [file 404_2025_8262_MOESM3_ESM.tiff]

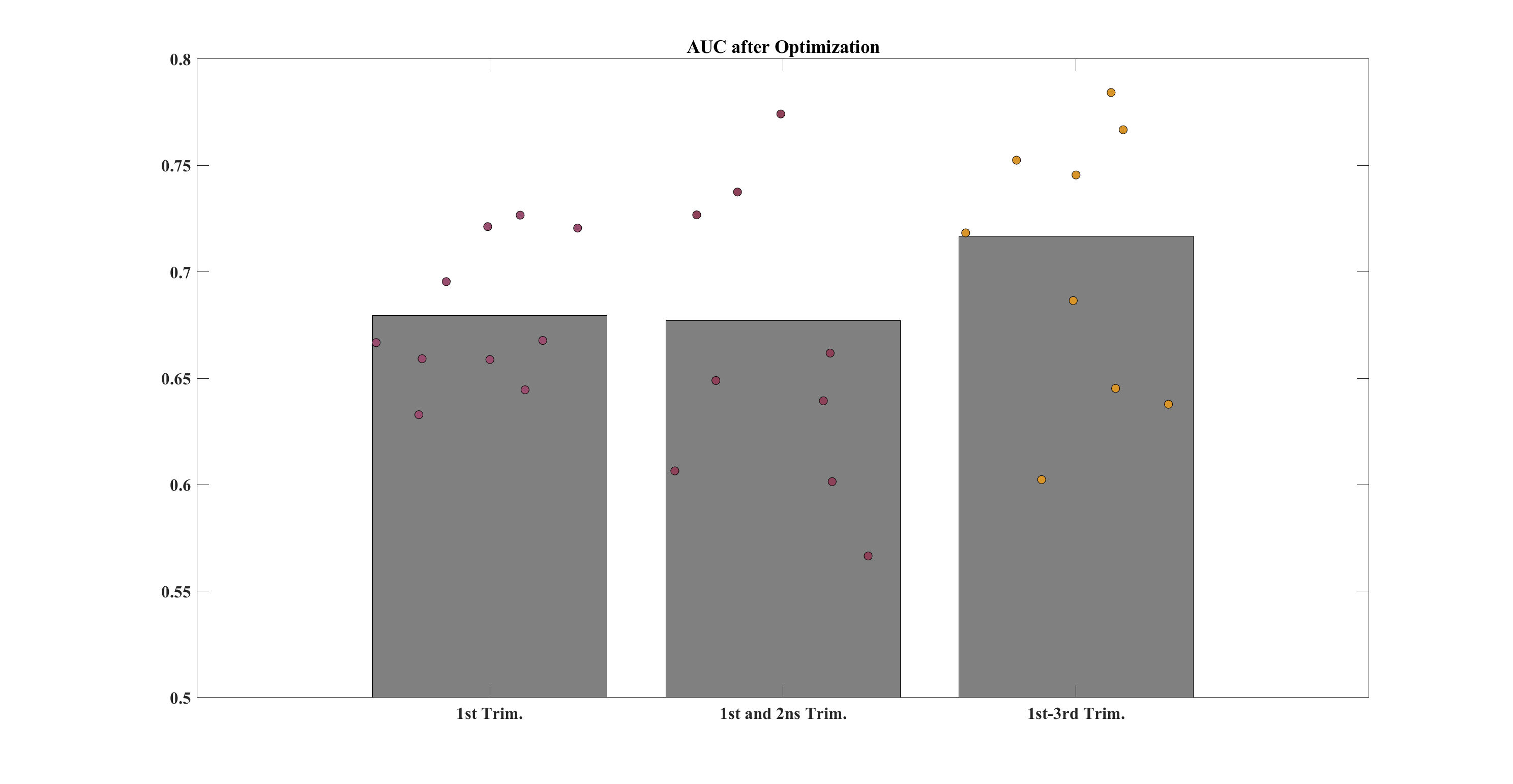

Supplement: Supplementary file 4 — Supplementary file4 (TIFF 272 KB) [file 404_2025_8262_MOESM4_ESM.tiff]
